# Imaging Analysis of Carbohydrate-Modified Surfaces Using ToF-SIMS and SPRi

**DOI:** 10.3390/ma3073948

**Published:** 2010-07-07

**Authors:** Kathryn M. Bolles, Fang Cheng, Jesse Burk-Rafel, Manish Dubey, Daniel M. Ratner

**Affiliations:** 1 Whitman College, 345 Boyer Ave., Walla Walla, WA 99362, USA; E-Mail: bolleskm@whitman.edu (K.M.B.); 2 Department of Bioengineering, University of Washington, Seattle, WA 98195, USA; E-Mails: ffcheng@u.washington.edu (F.C.); jbrafel@u.washington.edu (J.B.R.); 3 NESAC/Bio, University of Washington, Seattle, WA 98195, USA; E-Mail: mdubey@lanl.gov (M.D.)

**Keywords:** carbohydrate microarray, ToF-SIMS, SPR, glycomics

## Abstract

Covalent modification of surfaces with carbohydrates (glycans) is a prerequisite for a variety of glycomics-based biomedical applications, including functional biomaterials, glycoarrays, and glycan-based biosensors. The chemistry of glycan immobilization plays an essential role in the bioavailability and function of the surface bound carbohydrate moiety. However, the scarcity of analytical methods to characterize carbohydrate-modified surfaces complicates efforts to optimize glycan surface chemistries for specific applications. Time-of-Flight Secondary Ion Mass Spectrometry (ToF-SIMS) is a surface sensitive technique suited for probing molecular composition at the biomaterial interface. Expanding ToF-SIMS analysis to interrogate carbohydrate-modified materials would increase our understanding of glycan surface chemistries and advance novel tools in the nascent field of glycomics. In this study, a printed glycan microarray surface was fabricated and subsequently characterized by ToF-SIMS imaging analysis. A multivariate technique based on principal component analysis (PCA) was used to analyze the ToF-SIMS dataset and reconstruct ToF-SIMS images of functionalized surfaces. These images reveal chemical species related to the immobilized glycan, underlying glycan-reactive chemistries, gold substrates, and outside contaminants. Printed glycoarray elements (spots) were also interrogated to resolve the spatial distribution and spot homogeneity of immobilized glycan. The bioavailability of the surface-bound glycan was validated using a specific carbohydrate-binding protein (lectin) as characterized by Surface Plasmon Resonance Imaging (SPRi). Our results demonstrate that ToF-SIMS is capable of characterizing chemical features of carbohydrate-modified surfaces and, when complemented with SPRi, can play an enabling role in optimizing glycan microarray fabrication and performance.

## 1. Introduction

Advances in synthetic carbohydrate chemistry have enabled the creation of complex carbohydrate-modified surfaces for biomedical and glycobiology research [1,2,3,4]. The printed carbohydrate microarray stands as one of the most significant tools to arise from these synthetic methodologies. As with all glycan-modified surfaces, carbohydrate microarrays are prone to confounding effects of surface chemistry, heterogeneity and glycan bioavailability [5]. The paucity of analytical methods to characterize glycan surface chemistries has thus far necessitated that surface chemistries be deduced purely from bioactivity (e.g., protein binding). However, this approach lacks specific information about surface resident chemical species and molecular conformations at the interface [6,7,8]. Consequently, efforts to optimize array surface chemistries and design new biocompatible surfaces for glycomics applications have proceeded slowly.

Carbohydrates are vital to a myriad of biological processes, including cell recognition, adhesion, and signaling; and pathogen binding [6,9,10]. Found on cell surfaces and at biological interfaces, glycans – often in the form of glycoconjugates such as glycoproteins and glycolipids – are key mediators of both low- and high-affinity binding interactions. This pervasive role in biology makes glycomics [11], the study of carbohydrates in biological systems, an area of importance to subjects as diverse as protein folding and function, biomaterials and tissue engineering, vaccine design, host-pathogen interactions, and developmental biology [12]. However, glycomics-driven advances in these fields have been hindered by the need for significant quantities of pure carbohydrate, which are difficult to obtain in large quantities via either synthetic or natural routes (due to their synthetic and structural complexity). Overcoming these challenges demands new tools for glycomics that can minimize the consumption of precious synthetic or isolated materials while advancing research into this structurally diverse class of biomolecule.

In order to effectively probe the broad diversity of glycoconjugate variants, glycomics researchers have adopted the microarray paradigm that has been highly successful in genomics and proteomics research. The microarray platform provides high-throughput screening of a large variety of glycans against protein, nucleic acid or pathogen binding partners—affording an efficient method to investigate glycan-mediated interactions occurring at biological interfaces.

A common method of array fabrication is to print amino-modified glycans onto organic substrates bearing amine-reactive chemistries (e.g., *N*-hydroxysuccinimide ester, NHS) [3, 13, 14]. Formation of an amide linkage to the surface provides a stable display of the carbohydrate moiety. Traditionally, arrays were then evaluated against a simple panel of fluorescence-labeled carbohydrate-binding proteins with known binding specificities, but this method did not have well-established protocols for translating these results into quantifiable information on array surface chemistry and performance. This meant that until recently the quality of the printed glycan array was largely an unknown entity. To address this issue, improved quality control methods have been deployed to characterize printed arrays. For instance, immobilized glycan densities have been evaluated using quantifiable fluorescent linkers [13] and sophisticated fluorescence-based image analysis has been used to interrogate the quality of array printing conditions [15]. Certain features are commonly observed when performing image analysis on microarray spots or “elements”. With fluorescence-based imaging, hollow spots (“craters”) and rings (“coffee stains”) are often observed and attributed to suboptimal printing conditions or artifacts associated with fluorescence quenching of the probe. These artifacts present a challenge to fluorescence-based quantification and data interpretation.

This study utilizes complementary surface analytical techniques – Time-of-Flight Secondary Ion Mass Spectrometry (ToF-SIMS) and Surface Plasmon Resonance imaging (SPRi) – to obtain both chemical and biological information from carbohydrate-modified surfaces and visualize the distribution of surface species and their correlation to bioactivity. Drawing upon previous X-ray Photoelectron Spectroscopy (XPS) characterization of the effect of density on protein binding to glycan-modified surfaces [5], this study highlights the prominent role surface analysis can play in advancing new tools – such as the carbohydrate microarray – for the field of glycomics.

**Figure 1:**
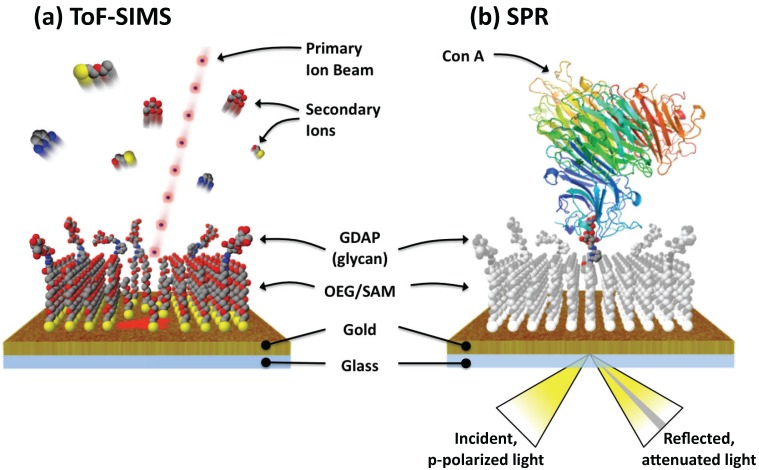
Analysis of glycan-functionalized array surfaces. **(a)** ToF-SIMS analysis generates a mass spectrum of the outermost 1.5 – 2.0 nm of a surface by bombarding it with a primary ion beam. In the case of carbohydrate-modified self-assembled monolayers on gold, the SIMS process generates ions that can be traced back to the underlying surfaces, the immobilization chemistries, and the glycans, generating a molecular image of the surface. **(b)** In SPR, real-time refractive index changes at the surface (*i.e.*, due to protein-carbohydrate binding, as shown) can be quantified and used to determine binding kinetics. This illustration shows the binding of the plant lectin Concanavalin A (Con A) to a carbohydrate-modified self-assembled monolayer (SAM).

ToF-SIMS is an ultra-sensitive surface analysis technique, probing the molecular composition of a surface with high mass resolution of the outermost ~1.5-2.0 nm. In ToF-SIMS, a primary ion beam strikes a surface, causing molecular fragments (secondary ions) to be generated (Figure 1a). The secondary ions are accelerated towards a detector, which determines their mass based on the time they take to reach the detector (hence “time-of-flight”). The imaging function of ToF-SIMS is achieved by rastering the primary ion beam across the surface. By focusing the primary ion beam, it is possible to resolve features down to sub-micrometer spatial resolutions, which can provide a chemical correlation to biological events occurring at the biointerface of a material [16,17]. Due to the volume of data generated by ToF-SIMS imaging, it is often necessary to use multivariate analysis (principal component analysis, PCA) to mine the data for ion species of interest and their corresponding locations. Not surprisingly, in the case of printed carbohydrate arrays, the location of glycan-derived secondary ions closely correlates to the spot where the carbohydrate was printed, while the chemistry of the underlying substrate is primarily found outside the printed element [17].

While ToF-SIMS provides a highly detailed view of the chemical composition of printed arrays, observed chemical species may not directly correlate to biological activity. For instance, variable density, suboptimal molecular orientation and adventitious surface contaminants can render arrayed biomolecules inactive, even though they are present on a surface. Therefore, a complementary method is required to determine bioactivity.

SPR is a powerful method for observing biomolecular interactions at an interface. Specifically, SPR measures changes in the refractive index close to a surface, providing quantitative binding kinetics (association/dissociation of a soluble binding partner) [18], and can detect low-affinity interactions. This makes SPR ideal for probing the bioactivity of model glycoarrays and carbohydrate-modified surfaces (Figure 1b). SPR is also able to provide real-time results without the need for fluorescent probes or labels. This simplifies experimental conditions and permits the detection of binding across a large dynamic range of bioactivity [7].

**Scheme 1:**
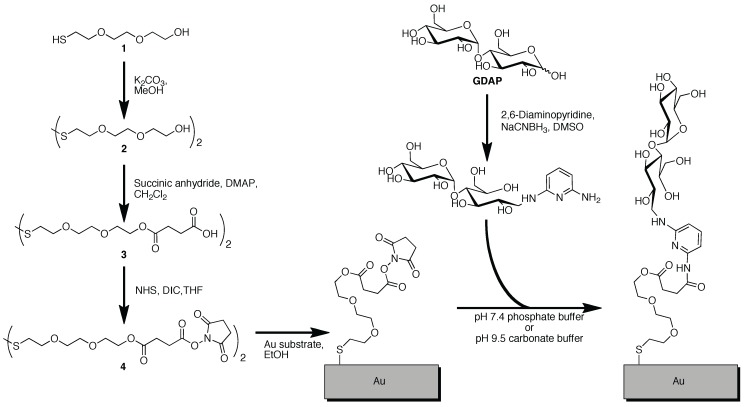
Synthesis of amine-reactive surfaces for the immobilization of amino-functionalized carbohydrates (*i.e.*, GDAP).

This study demonstrates the complementary application of ToF-SIMS and SPR imaging analysis to examine glycoarray surface chemistries and bioactivity. The primary objective is to establish biophysical surface analysis methods for glycan microarrays, enabling future investigations into the impact of surface chemistry on biological activity within the printed carbohydrate array. Carbohydrate microarrays were fabricated by printing a diaminopyridine-modified glycan (GDAP, see Scheme 1 in the Experimental Section) onto a model surface consisting of an NHS-activated oligoethylene glycol self-assembled monolayer (OEG/SAM). For this preliminary study, self-assembled monolayers (SAMs) were selected over commercial array substrates for their reliability, simple chemistries and extensive surface characterization by XPS, ToF-SIMs and SPR. Individual spots on the arrays were analyzed with ToF-SIMS imaging, generating peak lists for image interpretation. Peaks of interest were determined using PCA and the distribution of select molecular species was visualized and compared to SPRi bioactivity. This combined chemical- and biological-imaging approach provides insight into the factors that affect carbohydrate microarray performance.

## 2. Results and Discussion

### 2.1. ToF-SIMS Imaging and Principal Component Analysis

**Figure 2:**
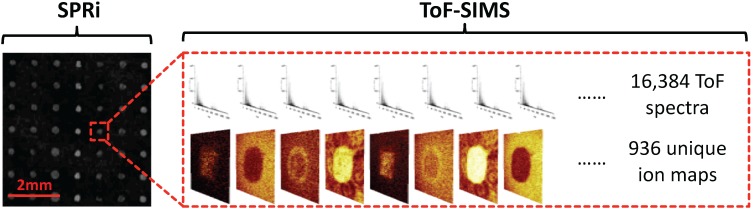
ToF-SIMS imaging analysis of a printed carbohydrate microarray—SPRi image (left) illustrates a carbohydrate-binding protein adsorbed to a printed glycan array; ToF-SIMS image analysis (right) represents acquisition of SIMS data from a printed spot. A 500 × 500 μm area (centered on an array spot) is rastered by a primary ion beam, generating 16,384 mass spectra, each corresponding to a ~3.9 × 3.9 μm pixel. These mass spectra can be combined to create ion maps (images) displaying the spatial abundance of each observed ion. In the case of the carbohydrate microarray, 936 unique ions were observed in a representative spot on the array.

ToF-SIMS images were acquired for individual spots on the array surface using 500 × 500 μm imaging windows (Figure 2). Each acquisition rastered the imaging window at a resolution of 3.9 μm per pixel, generating a 128 × 128 array of mass spectra (16,384 total). A typical spectrum (m/z from 1 through 350) contains more than nine hundred peaks having ion counts at least three times greater than background. The relative intensities of these peaks are interrelated, since they come from the same surface species. However, variation between printed glycan spots and the surrounding chemistries can be difficult to assess due to the volume of mass spectral data.

**Figure 3:**
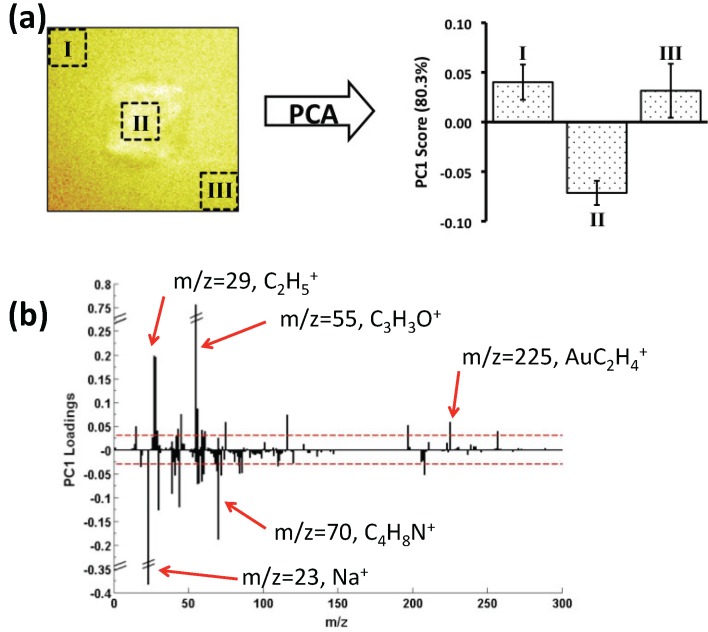
Principal component analysis (PCA) of a representative ToF-SIMS image. **(a)** PCA analysis is performed using three ROIs defined within a ToF-SIMS image (I, II, III), generating scores according to the principal components of the dataset. In the case of the representative ToF-SIMS image shown, ROIs I and III grouped together with positive loadings in PC1, while ROI II (corresponding to the printed glycan array element) had negative PC1 loadings. These results are consistent with the expected similarities and differences of the selected ROIs. **(b)** PC1 loadings were calculated for the observed SIMS ions, where positive loadings correspond to non-glycan features on the array (OEG/SAM and gold substrate) and negative loadings are attributable to the immobilized glycan (GDAP). An arbitrary PC1 threshold of ±0.03 (dotted red line) was used to select ions for further imaging analysis.

We employed a multivariate analysis approach to extract useful information from a dataset consisting of five image samples containing 16,384 spectra within each sample. In particular, chemical variation between the array spots and the surrounding surface was assessed by principal component analysis (PCA) [14]. Three 100 × 100 μm regions of interest (ROIs) were selected (upper left (I), center (II), and bottom right (III)) for each of 5 total ion images to produce 15 averaged spectra (Figure 3a). The 15 spectra were analyzed and the dataset was decomposed into scores and loadings using PCA. Scores give the quantitative relationships between sample spectra in the principal component-based axis system, while loadings give the quantitative relationship between the original mass peaks and the generated principal components. The scores and loadings for principal component 1 (PC1) are plotted in Figure 3. PC1 captured 80.3% of the total variation among spectra and distinguished region II from regions I and III (p < 0.0001) due to large differences between the glycan/spot (II) and substrate/background (I and III). Regions I and III were statistically similar (p < 0.2), indicating homogeneity of the background surrounding the spot. Positive PC1 peaks are attributed to substrate/background, while negative PC1 peaks arise from glycan related species. By thresholding peaks with largest PC1 loadings, ions contributing most to the differences between the glycan/spot and substrate/background can be identified. These select ions (those with PC1 loadings greater than ±0.03, see Figure 3b) and their peak assignments are listed in Table 1. All peaks were assigned to five categories of fragments: hydrocarbon, buffer salt, GDAP (glycan), OEG/SAM, and gold substrate (e.g., C_2_H_5_^+^, Na^+^, CH_4_N^+^, C_2_H_5_O^+^, and AuC_2_H_4_^+^, respectively). Fragments originating from hydrocarbon, salt, OEG/SAM, and gold substrate were consistent with previous reports [17,18,19,20]. Hydrocarbon and salt are well-known adventitious contaminants from printing and sample handling, and were therefore removed from the select peak list used in further data analysis.

**Table 1:** Select peaks from PCA and their assignments.

m/z	Fragment	Peak assignment
15.02	CH_3_^+^	Hydrocarbon
18.04	NH_4_^+^	GDAP
22.99	Na^+^	Buffer salt
27.02	C_2_H_3_^+^	Hydrocarbon
28.03	C_2_H_4_^+^	Hydrocarbon
29.00	CHO^+^	OEG/SAM
29.04	C_2_H_5_^+^	Hydrocarbon
30.04	CH_4_N^+^	GDAP
38.96	K^+^	Buffer salt
41.04	C_3_H_5_^+^	Hydrocarbon
43.02	C_2_H_3_O^+^	OEG/SAM
44.05	C_2_H_6_N^+^	GDAP
44.98	CHS^+^	OEG/SAM
45.03	C_2_H_5_O^+^	OEG/SAM
55.02	C_3_H_3_O^+^	OEG/SAM
56.03	C_3_H_4_O^+^	OEG/SAM
56.06	C_3_H_6_N^+^	GDAP
59.00	C_2_H_3_S^+^	OEG/SAM
59.05	C_3_H_7_O^+^	GDAP
60.06	C_2_H_6_NO^+^	GDAP
61.01	C_2_H_5_S^+^	OEG/SAM
69.04	C_3_H_5_N_2_^+^	GDAP
70.07	C_4_H_8_N^+^	GDAP
72.09	C_4_H_10_N^+^	GDAP
75.04	C_3_H_7_O_2_^+^	OEG/SAM
84.05	C_4_H_6_NO^+^	GDAP
84.09	C_5_H_10_N^+^	GDAP
99.06	C_5_H_7_O_2_^+^	OEG/SAM
110.09	C_6_H_10_N_2_^+^	GDAP
116.05	C_5_H_8_O_3_^+^	OEG/SAM
196.97	Au^+^	Gold substrate
225.00	AuC_2_H_4_^+^	Gold substrate
256.98	AuC_2_H_4_S^+^	Gold substrate

### 2.2. Visualization of Chemical Species within the Printed Glycan Array

**Figure 4:**
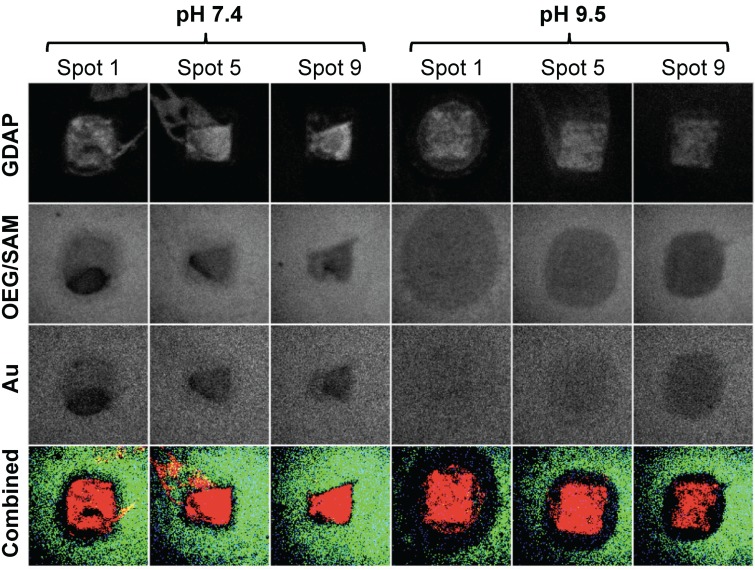
ToF-SIMS image analysis of three array elements, printed at pH 7.4 or 9.5. Spots 1, 5, and 9 were chosen for analysis because they represented the beginning, middle, and end of a sequence of microarray printing, respectively. Using the peaks identified by PCA (see Table 1), ion images corresponding to glycan (GDAP), the underlying SAM chemistry (OEG/SAM) and the gold substrate (Au) illustrate the distribution of chemical species around individual printed spots. Combined ion images using red for GDAP, green for OEG/SAM, and blue for Au illustrate their co-distribution. Ion images also illustrate the inhomogeneity of the printed spots, artifacts from the wash conditions, and the impact of pH on spot morphology.

The spatial distribution of GDAP, OEG/SAM, and gold was visualized to illustrate the chemical morphology of each array element. Identified surface species were grouped according to their source to generate ion maps corresponding to GDAP, OEG/SAM, and gold (Figure 4). Ion counts were normalized for visualization purposes. It should be noted that ToF-SIMS is not a quantitative method for determining surface composition, and ion count cannot be related directly to the absolute abundance of molecules at the surface. Furthermore, PCA-based data mining of ions, as represented in Figure 4, illustrates only a subset of the total ions derived by SIMS analysis and must be viewed as representative of differential distribution. Therefore, useful information on the location of molecular species at the surface can be derived by summing the ion counts for selected categories of fragments (GDAP, OEG/SAM and gold substrate). A binary threshold was subsequently applied to each ion image and the three sets of surface species were colorized (red for GDAP, green for OEG/SAM, and blue for gold substrate) and combined to generate a chemical map of the glycan array surface (Figure 4, Combined). ToF-SIMS images for varying printing conditions (printing sequence and pH) were examined with this visualization method (Figure 4). From left to right, the first three images and second three images were spots printed using pH 7.4 and pH 9.5 buffer solutions, respectively. In the array, the first, fifth, and ninth spots in a sequence of contact spotting were visualized by ToF-SIMS. As expected, GDAP was located in the center of the array element and surrounded by OEG/SAM. A boundary artifact between GDAP and OEG/SAM appeared prominently in the set of pH 9.5 spots. Such an artifact may be attributable to surface hydrolysis of the NHS-ester and/or oxidative damage to the self-assembled monolayer. Additional experimentation is required to explore these possibilities.

**Figure 5:**
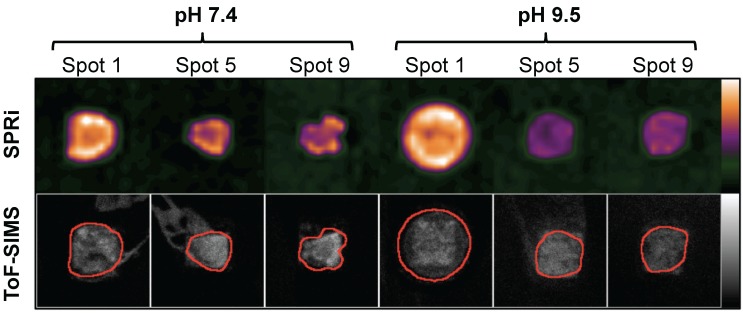
SPRi and ToF-SIMS analyses correlate glycan bioactivity and distribution between replicate spots on identical arrays, despite variability in spot morphology due to printing conditions. These results suggest that ToF-SIMS can be used to characterize printed glycan arrays and predict their bioactivity.

These images also display a degree of variability in spot size, shape, and homogeneity. A contributing factor to this variability is the propensity of the contact printing method to deposit decreasing amounts of liquid during sequential spotting, causing a declining trend in spot size. In addition, evaporation of the nanoliter volumes of printing buffer during glycan immobilization leads to microheterogeneity within array spots, as evidenced by characteristic “coffee stain” artifacts (Figure 5, SPRi Spot 1, pH 7.4 and 9.5). Finally, rinsing conditions following printing were observed to spread spots in the direction of flow, causing the “tail” effect (Figure 4, GDAP Spot 5, pH 7.4 and 9.5).

### 2.3. Comparison of Glycan Bioactivity and Molecular Species Distribution on Array Surfaces

SPRi protein binding images and ToF-SIMS glycan images were compared to determine whether the location of secondary ions associated with immobilized glycan corresponded to their bioactivity (Figure 5). The carbohydrate-binding protein (lectin) Concanavalin A (Con A) was used to probe for bioavailable glycan on the array surface. Con A has a strong affinity for non-reducing α-mannose and α-glucose residues; GDAP displays a terminal α-glucose. Regions of protein binding in the printed glycan spot were outlined by applying a threshold to the SPR images and overlaid on the ToF-SIMS GDAP images. SPRi and ToF-SIMS images were obtained from duplicate array surfaces fabricated in parallel using the same printing conditions and sequence.

Figure 5 demonstrates the correlation between ToF-SIMS identified GDAP and SPRi bioactivity. Both visualization techniques show that spots do not have consistent shape, glycan distribution, or bioactivity. However, the correlation between GDAP surface distribution and bioactivity remains remarkably strong, suggesting that these complementary methods are observing interrelated surface phenomena. These results represent the first time that the distribution of glycan on a substrate has been explicitly linked to bioactivity.

An important question is whether the differences observed between ToF-SIMS and SPRi surface features reflect real phenomena. For example, the “tail” artifact (visible in Figure 4 and Figure 5, Spot 5, pH 7.4 and 9.5) was visible in ToF-SIMS and not in SPRi. We believe this artifact is the product of rinsing during array fabrication. A “crater” or “coffee stain ring” can also be observed under SPRi that is occasionally, but not always, observed in ToF-SIMS. It is possible that the arrays used for these two analyses experienced slight differences in the printing and washing steps or that these artifacts are only visible under one of the visualization techniques. It should be noted that the ring artifacts (“coffee stains”), which are clearly visible within the SPRi images, are often observed during microarray printing. Other groups have investigated the cause of these rings [21,22], and we believe their prominence with the SPRi images is due to the significant role of glycan density during protein binding. Ongoing efforts are exploring how these glycan surface density effects can be controlled within printed arrays [5].

While it would be ideal to perform ToF-SIMS and SPRi on the same array, this was not possible in this study: ToF-SIMS is a destructive process that cannot be performed prior to SPRi and SPRi leads to protein contamination if performed prior to ToF-SIMS. Additionally, SPRi arrays were blocked with bovine serum albumin (BSA) prior to Con A binding to reduce non-specific binding of lectin, so ‘*in situ*’ analysis of lectin-binding by ToF-SIMS would be challenging. Use of duplicate arrays, fabricated in parallel, served in this study as a practical solution to explore both chemical and biological properties of these carbohydrate-modified surfaces. In the future, a possible solution would be to analyze a given array surface using low-dose ToF-SIMS, followed by SPRi analysis of the same substrate. Alternatively, fluorescence imaging could obtain information from labeled glycans and proteins.

### 2.4. SPR Region of Interest Selection and Response

**Figure 6:**
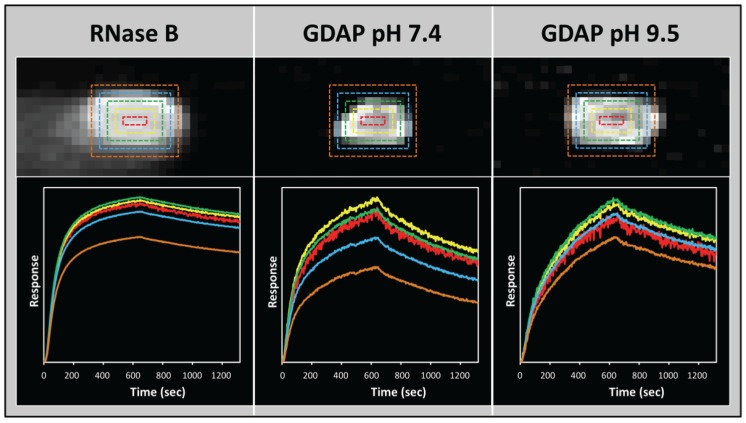
Influence of user ROI selection on SPRi results for Con A binding to printed RNase B (glycoprotein), GDAP pH 7.4, and GDAP pH 9.5 spots. (Top) SPRi image showing five concentric ROIs selected around the center of each printed glycan array element—printed spots range from 150-250 microns in diameter. (Bottom) Sensorgrams associated with each concentric ROI (red, yellow, green, blue and orange) show non-uniform response across the printed array element, and significant decreases in signal associated with large ROIs that extend beyond the spot boundary.

Effective interrogation of carbohydrate arrays with ToF-SIMS and SPR requires significant user input, which can impact post-acquisition interpretation of results. For instance, the influence of user ROI selection on SPR response was examined for three different spots (Figure 6). ROI selection had a profound effect on the observed response of Con A binding to printed RNase B (a glycoprotein that contains a natural high-mannose carbohydrate structure), GDAP pH 7.4, and GDAP pH 9.5 spots. Selecting large ROIs depressed responses in all three spots due to the inclusion of the non-glycan background. Conversely, small ROIs produced highly variable results due to their vulnerability to array element inhomogeneity, which was pronounced in the binding curves of the GDAP spots. It should be noted that Con A has a higher affinity for α-mannose residues and that dissociation of Con A from the branched mannoside of RNAse B is significantly lower than that of GDAP—this is predicted by Con A’s carbohydrate-binding specificity.

**Figure 7:**
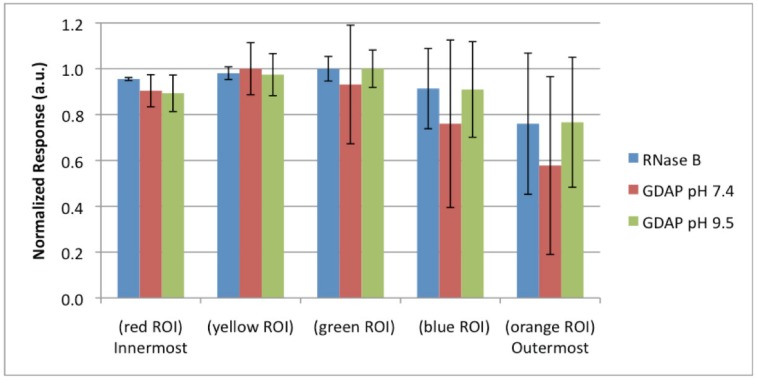
Normalized SPR response as a function of increasing ROI area (corresponding to SPRi images from Figure 6). SPR response was measured near maximal binding (11 min) and was highest for ROIs fully contained within the printed spots. Standard deviation of SPR response increases dramatically for ROIs that include a larger area.

We quantified the effect of ROI selection on SPR response and variability. Using the ROIs in Figure 6, significant trends appeared in both the intensity and standard deviation of the SPR response (Figure 7). A strong positive correlation can be observed between the size of the ROI and the standard deviation. The outermost (orange) ROIs exhibited depressed SPR response. The green and yellow ROIs, the largest contained entirely within the printed spots, had the highest response. These trends were consistent across all three spots, reinforcing the importance of ROI selection in achieving comparable and optimized responses.

As carbohydrate arrays move towards higher printing densities with smaller spots placed more closely together, microheterogeneity within individual spots will have a more significant effect in array results. Investigators utilizing SPRi must recognize the importance of ROI selection since we have shown that SPR response can be significantly altered through user manipulation of data. Improvement in spot homogeneity and SPRi resolution may alleviate the impact of ROI selection on response. In the case of this study, an acceptable SPR response was observed when the ROI selection was within the borders of the spot, avoiding artifacts at the edge while including as much of the printed area as possible.

## 3. Experimental Section

### 3.1. Reagents and Materials

All chemical reagents were purchased from Sigma-Aldrich (St. Louis, MO) and Acros Organics (West Chester, PA) and were used as received unless specified. Bovine serum albumin (BSA) was purchased from Sigma-Aldrich (St. Louis, MO). Concanavalin A (Con A) was purchased from MP Biomedicals (Solon, OH). Ethanol (200 proof, USP) was purchased from Decon Labs (King of Prussia, PA). SF-10 glass substrates were purchased from SCHOTT Glass Technology (Duryea, PA). Silicon wafers were purchased from Silicon Valley Microelectronics (San Jose, CA).

*1-Mercaptotriethylene Glycol* **(1)***.* The synthesis of **1** has been reported elsewhere [23]. Briefly, hydrochloric acid in ethanol (1:5 v/v) was added dropwise to a solution of triethylene glycol monochlorohydrin (10.0 g, 0.06 mol) and sodium hydrogen sulfide (16.6 g, 0.30 mol) in ethanol at 60 °C. After the reaction was complete, ethanolic hydrochloric acid was added to quench the remaining hydrogen sulfide. The precipitated solid was removed by filtration and the ethanol was evaporated. The residue in dichloromethane was washed with bicarbonate, water, and brine. The organic phases were dried over MgSO_4_ and concentrated *in vacuo* to yield a colorless oil (7.6 g, 76%). ^1^H-NMR (CDCl_3_): δ 1.50 (s, 1H, -SH); 2.70 (b, 1H, -OH); 2.55 (t, 2H, -C*H*_2_-SH); 3.35-3.55 (m, 8H, -O-C*H*_2_-C*H*_2_-O-C*H*_2_-C*H*_2_-OH); 3.55-3.62 (m, 2H, -C*H*_2_-OH).

*Bis[8-hydroxy-3,6-dioxaoctyl]* *Disulfide* **(2)***.* Iodine (1.1 g, 4.5 mmol) in 50 mL methanol was added dropwise to a mixed solution of **1** (1.5 g, 9.0 mmol) in 50 mL methanol and 25 mL potassium carbonate aqueous solution (3% w/v) at room temperature. Several drops of sodium sulfide were added until the yellow color disappeared. The evaporated residue was suspended in ethanol and the potassium salt was filtered off. The resulting solution was evaporated again, chromatographed on a silica gel column using a gradient of CH_3_Cl/MeOH (100:0 → 90:10), and concentrated *in vacuo* to yield a clear yellow oil (1.2 g, 81%). ^1^H-NMR (CDCl_3_): δ 2.89-2.93 (t, 4H, -C*H*_2_-S-); 3.55-3.65 (m, 4H, -C*H_2_*-OH); 3.63-3.68 (m, 12H, -O-C*H*_2_-C*H*_2_-O-C*H*_2_-CH_2_-OH); 3.71-3.77 (m, 6H, -C*H*_2_-CH_2_-S-, -C*H*_2_-OH, -O*H*).

*Carboxylic disulfide* **(3)***.* Compound **2** (0.8 g, 2.4 mmol) was added to a mixed solution of 2,4-dimethylaminopyridine (1.0 g, 9.6 mmol) and succinic anhydride (1.2 g, 9.6 mmol) in anhydrous dichloromethane at room temperature. After 2 h stirring, the solution was evaporated and passed through a silica gel column using a gradient of CH3Cl/MeOH/acetic acid (100:0:0.1 → 90:10:0.1). The fractions were concentrated *in vacuo* to yield a yellow oil (1.2 g, 90%). ^1^H-NMR (CDCl_3_): δ 2.65-2.73 (m, 8H, -C*H*_2_-S- and -C*H*_2_-CH_2_-COOH); 2.85-2.95 (m, 4H, -CH_2_-C*H*_2_-COOH); 3.65-3.95 (m, 16H, -O*CH_2_*-*CH_2_*-O-CH_2_-, -S-CH_2_-*CH_2_*-O- and -O-C*H*_2_-CH_2_-O-CO-); 4.20-4.30 (t, 4H, -*CH_2_*-O-CO-).

*NHS disulfide* **(4)***. N*, *N*’-diisopropylcarbodiimide (150 mg, 1.2 mmol) was added to a mixed solution of **3** (250 mg, 0.5 mmol) and *N*-hydroxysuccinimide (135 mg, 1.2 mmol) in 1 mL anhydrous tetrahydrofuran at room temperature. After white precipitate appeared (2+ hours), the reaction solution was quickly passed through a silica gel column using a gradient of toluene/ethyl acetate (70:30 → 20:80). The fractions were concentrated *in vacuo* and passed through a second silica gel column using a gradient of toluene/ethyl acetate (70:30 → 40:60). The final fractions were concentrated *in vacuo* to yield a white oil (340 mg, 82%). ^1^H-NMR (CDCl_3_): δ 2.75 -3.00 (m, 20H, -C*H*_2_-S-, -O-COC*H*_2_-C*H*_2_-CO-O-, and –N-CO-C*H_2_*-C*H*_2_-CO-); 3.60-3.70 (s, 8H, -O-C*H*_2_-C*H*_2_-O-); 3.70-3.85 (m, 8H, -S-CH_2_-*CH_2_*-O- and -O-C*H*_2_-CH_2_-O-CO-); 4.25-4.35 (t, 4H, -*CH_2_*-O-CO-).

### 3.2. Glycan-diaminopyridine (GDAP)

The synthesis of GDAP has been reported elsewhere [13]. Briefly, maltose (5.8 mg, 16.1 μmol) was added to a mixed solution of 2,6-diaminopyridine (0.35 M) and sodium cyanoborohydride (1.0 M) and mixed in 0.2 mL dimethyl sulfoxide/acetic acid (7:3, v/v). The mixture was incubated at 65° C for 4 h and evaporated *in vacuo*. The resulting residue was dissolved in water and chromatographed on 3MM paper disks pre-treated with acetic acid. The paper disks were washed by 100% acetonitrile, 96% acetonitrile in water, and water. The aqueous fractions were lyophilized to yield a white solid (4 mg, 56%). ESI-MS: m/z 436.5 for [M+H]^+^ and m/z 458.2 for [M+Na]^+^.

*Preparation of gold substrate.* Titanium (2 nm) and gold (45 nm) films were deposited onto cleaned SF-10 glass (18 mm × 18 mm) using electron beam evaporation at Washington Technology Center.

*Amine-reactive surfaces.* Fresh gold substrates were immersed in NHS-disulfide (**4**, saturated solution in absolute ethanol) for 2 h at room temperature to form self-assembled monolayers (SAMs). After SAM formation, the surfaces were dipped in a stirring ethanol bath for 1 min. The cleaned surfaces were gently dried with a stream of argon and stored in a vacuum desiccator. This method generates high quality and reproducible SAMs (as determined by XPS) bearing NHS with excellent surface homogeneity.

*Array printing.* Freshly prepared amine-reactive surfaces were placed into a SpotBot2 microarrayer (TeleChem International, Inc.) and GDAP and RNase B were printed in 46% relative humidity (R. H.) using a SMP8 Stealth pin, creating a 10 × 10 array. GDAP was printed at a concentration of 1 mM in selected buffer solutions. RNase B was printed at approximately 1 mg/mL. Printed microarray chips were allowed to sit for 2 h in the array printer and transferred to a 75% R. H. chamber overnight. The chips were removed and immersed in PBS containing 0.5% BSA and 0.1% Tween-20 (PBS-BT) for 30 min at room temperature, then rinsed with ultrapure water and dried under a stream of argon.

*SPRi.* SPRi was performed on an SPRimagerII from GWC Technologies. The SPRimagerII was operated at room temperature using a standard flow cell and a peristaltic pump (BioRad-EconoPump) at 100 μL/min. All surfaces were passivated with PBS-BT for 30 min and equilibrated in PBS prior to protein binding. Data acquisition consisted of the averaging of 30 images over a short duration to create an average image and the response was displayed in pixel intensity units. Urea (8 M) was used to strip bound protein and regenerate the array surfaces. For sensorgram acquisition, a variable region-of-interest (ROI) was selected, ranging from 50 × 50 μm to 200 × 200 μm. For purposes of presentation, the contrast and brightness of SPR images were adjusted using ImageJ software (U. S. National Institutes of Health, Bethesda, MD).

*ToF-SIMS image.* Data for microarray surfaces were acquired on an ION-TOF 5–100 instrument (ION-TOF GmbH, Münster, Germany) using a Bi_3_^+^ primary ion source. Positive ion images were acquired with a pulsed 25 keV, 1.3 pA primary ion beam in high current bunched mode (*i.e.*, high mass resolution mode) from 500 × 500 μm areas on simple array elements on the sample surfaces. All images contained 128 × 128 pixels. These analysis conditions resulted in spatial resolution of approximately 3.9 μm. All data were collected using an ion dose below the static SIMS limit of 1×10^12^ ions/cm^2^. A low-energy electron beam was used for charge compensation on the microarray samples. The mass resolution (m/∆m) of the positive secondary ion spectra was typically above 6000 for the m/z = 27 peak.

*PCA spectra analysis.* ToF-SIMS spectra were generated based on 100 × 100 μm ROIs selected in the upper left, center, and lower right regions of the 500 × 500 μm total ion image (Figure 3a). All of the peaks greater than or equal to three times the value of the background in the 1-350 m/z region of the ROI spectra were selected for principal component analysis. These peaks were mean centered and normalized to the total selected ion intensity. PCA was then performed on this dataset using a series of scripts written by NESAC/BIO for MATLAB (MathWorks, Inc., Natick, MA). By applying PCA, a ToF-SIMS dataset can be reduced to two cross-product matrices: scores and loadings. Scores plots can be used to visualize the quantitative relationship between samples. Loadings plots can be used to visualize the relationship between original variables (spectral peaks) and new variables (principal components).

## 4. Conclusions

In order for the carbohydrate microarray to realize its full potential as a tool for biomedical research and drug discovery, the microarray’s biointerface must be rigorously interrogated and optimized to account for the effects of surface inhomogeneity, printing artifacts, and adventitious contaminants. This study demonstrates the application of ToF-SIMS and SPR imaging to characterize carbohydrate array surface chemistries and bioactivity. ToF-SIMS analysis of carbohydrate-modified surfaces was accomplished through the use of principal component analysis to distinguish chemical variation between glycan spots and their surroundings, and to identify secondary ions associated with bioactive surface features. The distribution of glycan, OEG/SAM, and gold substrate was visualized and compared to SPRi protein binding images, providing valuable information for the future optimization of printing conditions (e.g., pH, rinsing). These results also highlight the importance of a user’s ROI selections during SPRi analysis of glycoarrays, as ill-considered ROIs alter the observed response and lead to increased variability. This study establishes the utility of ToF-SIMS and SPRi for carbohydrate microarray analysis. Combined with XPS, the application of these complementary analytical tools will further development of the microarray platform and provide investigators additional information to aid in the interpretation and validation of array results.
